# Pathogenesis of Endometriosis and Endometriosis-Associated Cancers

**DOI:** 10.3390/ijms25147624

**Published:** 2024-07-11

**Authors:** Altynay Adilbayeva, Jeannette Kunz

**Affiliations:** Department of Biomedical Sciences, School of Medicine, Nazarbayev University, 5/1 Kerey and Zhanibek Khans St, Astana 020000, Kazakhstan; altynay.adilbayeva@nu.edu.kz

**Keywords:** endometriosis, endometriosis-associated cancer, ovarian cancer, estrogen, PI3K/mTOR, ARID1A, KRAS, inflammation, organoids, single-cell RNA sequencing

## Abstract

Endometriosis is a hormone-dependent, chronic inflammatory condition that affects 5–10% of reproductive-aged women. It is a complex disorder characterized by the growth of endometrial-like tissue outside the uterus, which can cause chronic pelvic pain and infertility. Despite its prevalence, the underlying molecular mechanisms of this disease remain poorly understood. Current treatment options are limited and focus mainly on suppressing lesion activity rather than eliminating it entirely. Although endometriosis is generally considered a benign condition, substantial evidence suggests that it increases the risk of developing specific subtypes of ovarian cancer. The discovery of cancer driver mutations in endometriotic lesions indicates that endometriosis may share molecular pathways with cancer. Moreover, the application of single-cell and spatial genomics, along with the development of organoid models, has started to illuminate the molecular mechanisms underlying disease etiology. This review aims to summarize the key genetic mutations and alterations that drive the development and progression of endometriosis to malignancy. We also review the significant recent advances in the understanding of the molecular basis of the disorder, as well as novel approaches and in vitro models that offer new avenues for improving our understanding of disease pathology and for developing new targeted therapies.

## 1. Introduction

Endometriosis affects approximately 5–10% of reproductive-age women and significantly impacts quality of life due to debilitating pelvic pain and infertility [1,2,3]. This condition is characterized by the presence of endometrial tissue outside the uterus, most commonly on the ovaries, fallopian tubes, and pelvic peritoneum, but endometrial lesions have also been detected in the lungs, liver, pericardium, surgical scars, limbs, and even the central nervous system [1,2,3]. Like the tissue that normally lines the uterus, the hormone responsiveness of endometriotic tissue is maintained. Consequently, each month, the ectopic tissue responds to hormonal changes during the menstrual cycle, similar to that of the normal endometrium, by building up and shedding [1,2,3]. This, in turn, leads to cyclical bleeding inside the pelvis, chronic inflammation, and progressive scarring of the normal tissue surrounding the endometrial lesion.

Endometriosis is difficult to diagnose because symptoms can vary considerably among affected individuals. The most common symptoms of endometriosis include pelvic pain, especially excessive menstrual cramps (dysmenorrhea); pain during intercourse; abnormal or heavy menstrual flow; and infertility [3,4]. Other symptoms may include painful urination, bowel movements during menstruation, and gastrointestinal problems. Nevertheless, some patients may be asymptomatic. In particular, infertile patients often have no painful symptoms, and their disease is only diagnosed during the diagnostic work-up for infertility. The reason for the divergence in clinical manifestations is not known but may depend on the location of the ectopic tissue, the depth of infiltration, and the tendency of lesions to form scars or invade neuronal structures [5,6,7].

Currently, no universally validated diagnostic tests or screening tools that are specific to endometriosis are available. Often, a thorough evaluation of menstrual symptoms and chronic pelvic pain form the basis for suspecting the condition. However, as not all patients experience pain, infertility may be the first sign of the disease. The only definitive way to diagnose endometriosis requires surgical diagnosis or laparoscopy, which leads to a 7- to 11-year-long latency period from the onset of symptoms to definitive diagnosis [8] and carries an increased risk of disease progression within that period [9]. However, in recent years, noninvasive imaging methods, such as transvaginal ultrasound and magnetic resonance imaging (MRI), have improved the ability to diagnose endometriosis [10,11,12], allowing for the staging and classification of most forms of endometriosis without surgical intervention.

At the time of diagnosis, there are limited data on the optimal treatment options and short- or long-term prognoses. There is no cure for endometriosis, and treatment is generally aimed at controlling symptoms. Progesterone-based therapy has become the mainstay treatment for endometriosis, but progesterone resistance occurs in up to 40% of patients [3]. In addition, synthetic androgen therapy or gonadotropin-releasing hormone (GnRH) agonists, which induce a hypoestrogenic state, are prescribed to relieve pain from endometriosis, but, due to their side effects, these are not long-term options [3,5]. For many women, particularly those affected with endometriosis who do not respond to hormonal therapy, surgical removal can be extensive, requiring segmental bowel excision and extirpative resection of pelvic tissues. The recurrence of lesions over time is frequent, and patients may require additional surgery [13]. Endometriosis is thus associated with significant individual and public health costs, underscoring the importance of understanding the molecular mechanisms that underly disease pathogenesis and pathophysiology in order to develop effective noninvasive diagnostic tools and treatment options.

## 2. Histopathogenesis

**Figure 1:**
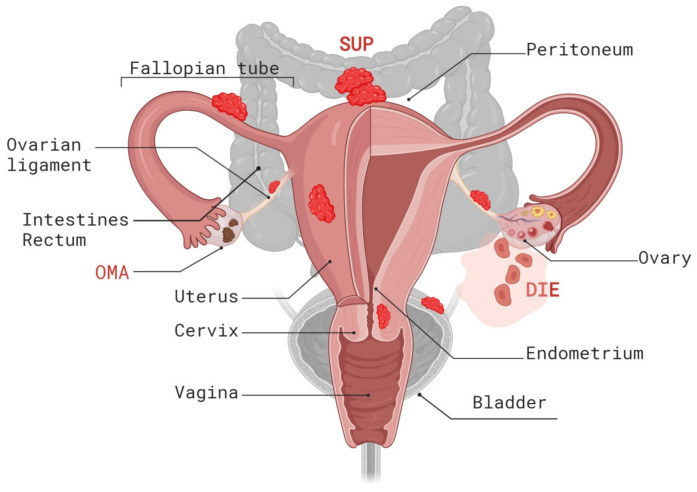
Types and locations of endometriotic lesions. Endometriosis is classified into three distinct subtypes: superficial peritoneal endometriosis (SUP), deep infiltrating endometriosis (DIE), and ovarian endometriosis (endometriomas, OMA). This figure was generated using Biorender.com (accessed on 18 June 2024).

Based on anatomical and histological criteria, endometriosis can be classified into three distinct subtypes: superficial peritoneal endometriosis (SUP), deep infiltrating endometriosis (DIE), and ovarian endometriosis (endometriomas, OMA) [3,4] (Figure 1).

SUP represents the most common type of endometriosis. These lesions involve the peritoneum, a thin layer covering the inner surface of the pelvic cavity. SUP lesions form flat, shallow patches that do not invade the underlying peritoneum. In contrast, DIE is a particular form of endometriosis that invades tissue (defined as lesions that infiltrate >5 mm under the peritoneal surface) and may have the ability to metastasize. Endometriomas are cystic masses that almost exclusively form deep within the ovaries and cases involving these account for up to 44% of women diagnosed with endometriosis [14]. These cysts are filled with old blood and, given the resulting color, are also referred to as “chocolate cysts”.

Several endometriosis classification systems exist, including the revised American Society for Reproductive Medicine (rASRM) scoring system and the ENZIAN classification for DIE [15,16]. More recently, the #ENZIAN classification was developed and published by J. Keckstein et al., which includes ovarian, peritoneal endometriosis, and pelvic adhesions and represents a comprehensive classification system for a complete mapping of endometriosis that reflects anatomical location, size of the lesions, adhesions, and degree of involvement of the adjacent organs [17]. These classification systems help document surgical, anatomical, and histological findings; however, the defined endometriosis subtypes poorly correlate with the severity of symptoms or the tendency to progress to malignancy. While patients with SUP can suffer from pelvic pain, OMA and DIE are the two forms of endometriosis most strongly associated with chronic pelvic pain and other symptoms, such as dysmenorrhea, deep dyspareunia, and painful defecation, and thus are considered more challenging to manage than SUP [3,18]. Moreover, of the three subtypes of endometriosis, only OMA is significantly associated with an increased risk for ovarian cancer [19]. Thus, while DIE is characterized by cancer-like local invasive behavior and the ability to metastasize, this subtype of endometriosis very rarely progresses to malignancy. Similarly, SUP rarely undergoes malignant transformation. Current classification systems for endometriosis are insufficient for describing the symptoms and prognosis of patients with different endometrial subtypes. Thus, developing a more biologically informed molecular disease classification incorporating the genetic and molecular characteristics of endometriosis will likely improve the diagnosis, risk assessment, and management of endometriosis.

## 3. Origin of Endometriosis

The origin of endometriosis is still contentious. Numerous models have been proposed to explain how endometriosis is initiated and how endometriotic tissue can grow throughout the abdominal cavity and disperse to extrapelvic locations [2,3,4].

**Figure 2:**
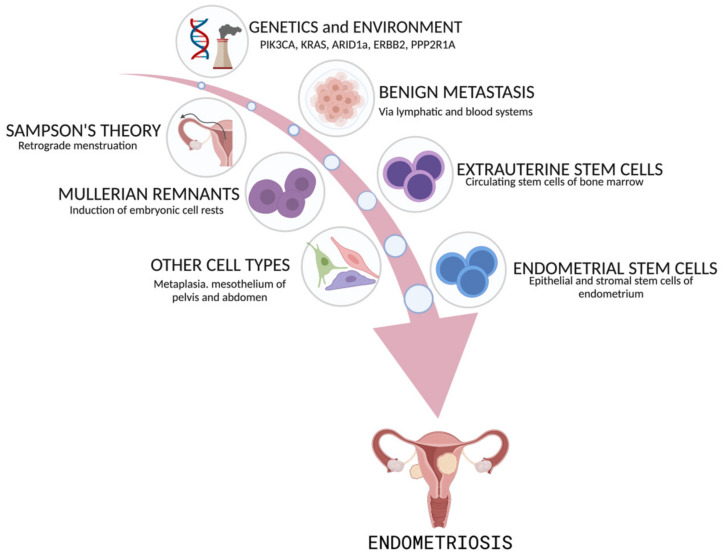
Theories regarding the pathogenesis of endometriosis. This figure was generated using Biorender.com (accessed on 18 June 2024).

However, none of these models can fully explain the full spectrum of the disease, and several different mechanisms may contribute to endometriosis development. Currently, retrograde menstruation or models involving a stem cell origin are the most intensely studied hypotheses. Other theories include benign metastasis through the hematogenous or lymphatic spread of endometrial cells, the transformation of the peritoneal mesothelium (called “coelomic metaplasia”), and the induction of Müllerian rest (Figure 2).

John Sampson is credited with developing the hypothesis that endometriosis arises from the efflux of eutopic endometrium (tissue fragments and cells) that is regurgitated into the peritoneal cavity during menstruation via the fallopian tubes [20]. Sampson’s theory of retrograde menstruation accounts for the observed physical displacement of endometrial tissue fragments into the peritoneal cavity during menstruation. This is widely accepted as a major contributor to endometriosis development. However, retrograde menstruation cannot explain the detection of endometrial tissue in extrapelvic locations and in rare cases of endometriosis that develops in the absence of a uterus, such as in men taking large doses of estrogen. Theories of the nonuterine origin of disease, such as coelomic metaplasia and Müllerian remnant differentiation, may explain these cases of endometriosis. Coelomic metaplasia involves the transformation of normal cells in the peritoneal lining to endometrial cells, possibly induced by hormonal or immunologic factors. This could explain the specific presence of lesions on the peritoneum [4]. Müllerian rest induction involves residual cells migrating from the embryologic Müllerian duct, maintaining their capacity to develop into endometriotic lesions under the influence of estrogen [20,21]. Finally, the theory of benign metastasis holds that ectopic endometrial lesions result from the lymphatic or hematogenous dissemination of endometrial cells [22].

Recently, the stem cell origin theory of endometriosis has received considerable attention due to advances in molecular and genetic research. There are two distinct models based on the stem cell’s tissue of origin: stem cells derived from the regenerating uterine endometrium or stem cells derived from the bone marrow. The uterus is the only organ that undergoes repeated cycles of shedding, repair, and regeneration over the course of a woman’s lifetime. The capacity for regeneration following menstruation is thought to depend on adult stem cells located at the endometrial/myometrial interface, also known as the endometrial functionalis layer [23]. This layer persists after menstruation and regenerates the endometrial epithelium during the proliferative phase in response to estrogen [24]. The first model of stem cell origin postulates that, during menstruation, circulating epithelial stem cells intended to regenerate the uterine endometrium are shed from the endometrial functionalis layer [24,25] and may become abnormally activated and trapped outside the uterus. This population of adult stem cells may then establish ectopic lesions following retrograde menstruation and trans-tubal migration into the pelvic cavity [26].

The second model of stem cell origin proposes that extrauterine stem/progenitor cells originating from bone marrow mesenchymal stem progenitors or endothelial progenitors may differentiate into endometriotic tissue (Figure 2) [24,27,28]. Support for a nonendometrial origin for endometriosis is derived from the detection of histologically confirmed endometriotic tissue in women with Rokitansky–Kuster–Hauser syndrome, a rare disorder that is characterized by the lack of a menstrual endometrium [29]. The stem cell origin of endometriosis may explain the clonality observed in many endometriotic lesions and is consistent with multiple existing theories of endometriosis origin since endometriosis can arise from endometrial or bone marrow stem cells dispersed via retrograde menstruation, via the hematogenous/lymphatic dissemination of stem cells or through persistence in Müllerian rests.

Recent advances in next-generation and single-cell RNA sequencing technologies, along with methods such as lineage tracing, tissue-clearing microscopy, and three-dimensional reconstruction, have enhanced our understanding of the architectural complexity and interconnectedness of endometrial glandular structures and basalis glands [30,31]. These investigations have illuminated the heterogeneity and diversity of cell types within the endometrium and endometriotic lesions and their origins. Multiple potential stem/progenitor cell subpopulations have been identified. Current theories propose that stem cells may arise from both the epithelial and stromal layers of the endometrium, with each stem cell type playing a unique yet cooperative role in the pathogenesis of endometriosis. This model, termed the dual stem cell theory [32], posits that two or more endometrial stem/progenitor cell types collaborate to form endometriotic lesions. Several progenitor endometrial cell subpopulations, predominantly but not exclusively located in the basalis endometrium, have been described. These cells exhibit stem cell properties, including self-renewal, differentiation, and high proliferative capacity [33]. Additionally, various candidates for initiating endometriotic epithelium have been identified. Notably, populations of endometrial epithelial progenitor cells, which test positive for N-cadherin (CDH2), stage-specific embryonic antigen-1 (SSEA-1), or both markers, are located at the bases of glands in the basalis layer [33,34,35]. Multiple research groups have identified another population of epithelial progenitor cells expressing the SRY-box transcription factor 9 (SOX9) and leucine-rich repeat-containing G-protein coupled receptor 5 (LRG5) stem cell markers has been identified by multiple research groups [33,34,35]. More recently, Tan et al. identified a distinct subset of progenitor cells positive for the mucin MUC5 through single-cell RNA sequencing studies. These cells have been observed in both the eutopic endometrium and ectopic lesions [36].

Candidate cells for initiating endometriotic stroma may be endometrial mesenchymal stem cells positive for Sushi domain containing 2 (SUSD2), platelet-derived growth factor receptor beta (PDGFRB), also termed cluster of differentiation 140b (CD140b), and CD146 [37,38]. These stroma-initiating cells are perivascularly situated within both the functionalis and basalis layers. In addition to these cell types, ongoing studies explore additional progenitor cells in the endometrium, such as endothelial progenitor cells located perivascularly in both the functionalis and basalis layers. In addition to these two types of stem/progenitor cells, there is ongoing research into other potential progenitor cell types in the endometrium, including endothelial progenitor cells [39,40], which might contribute to vascularization, as well as side population cells [41,42,43,44,45]. Furthermore, progenitor cells have been identified within a newly discovered rhizome network in the stratum basalis, which underlies the formation of interconnected endometrial glands with a potential shared origin [31].

Thus, abnormal endometrial stem/progenitor cells are likely the cellular origin of primary endometriotic lesions. These cells can either infiltrate the pelvic cavity and invade the mesothelium or disseminate via the bloodstream, leading to distant infiltration.

## 4. Risk Factors for Endometriosis

### 4.1. Genetic Risk Factors

Although the etiopathology of endometriosis still needs to be fully defined, a growing body of research suggests that heritable genetic factors play a vital role in the development of endometriosis and may help explain the different forms of endometriosis and disease manifestations. Familial clustering of the disease was noted as early as the 1950s [46], and the contribution of a genetic component to endometriosis was supported by many subsequent studies [47,48,49]. These studies have shown that first-degree relatives of women diagnosed with histologically confirmed endometriosis have a five-to-seven times greater risk of being affected by the disease [47,48]. In addition, familial endometriosis is typically associated with an earlier age of symptom onset and a more severe disease course than nonfamilial cases [50]. A genetic predisposition to endometriosis has been corroborated by twin studies showing that monozygotic twins are at increased risk of endometriosis compared with dizygotic twins [51].

Endometriosis is an estrogen-dependent disease, and estrogen and estrogen receptor (ER) signaling components play important roles in endometriosis pathogenesis [52]. Consistent with this notion, significant risk factors in all ethnicities include conditions that involve extended exposure to endogenous estrogens throughout life (early menarche or late menopause) and conditions that lead to variations in estrogen levels or affect menstruation, such as short menstrual cycle length, heavy menstrual bleeding, and uterine outlet obstruction [53]. Furthermore, population-based genealogy studies in various ethnicities have revealed a significantly increased risk of endometriosis linked to Asian ethnicity [54].

### 4.2. Modifiable Risk Factors

Diet and lifestyle modifications can significantly impact the risk of endometriosis by affecting estrogen levels, inflammation, the menstrual cycle, and prostaglandin metabolism [55]. Several studies have demonstrated that a diet rich in green vegetables, fresh fruits, and omega-3 and omega-6 polyunsaturated fatty acids is associated with a reduced risk of endometriosis [1,56]. In contrast, diets high in trans fats, coffee, and red meat have been linked to an increased risk of developing endometriosis [57]. Additionally, physical activity reduces the risk of developing many gynecological diseases, including endometriosis [55,58]. Furthermore, environmental factors, such as exposure to endocrine-disrupting chemicals, also contribute to endometriosis risk [55,59].

Cigarette smoking is negatively associated with the risk of endometrial cancer [60]. Consequently, numerous studies have examined the relationship between smoking habits and endometriosis risk, yielding inconsistent results. Some research indicates that smoking might reduce the risk of endometriosis [61,62,63,64,65], potentially due to its antiestrogenic effects [66]. Conversely, other studies have identified a positive association or found no significant relationship [64,67,68,69,70]. Similarly, the relationship between coffee or alcohol intake and endometriosis risk remains unclear. While some studies have suggested a protective effect against endometriosis [65,71,72], others have reported no clear association or opposite trends [55,70]. Recent studies have identified an association between changes in the composition of the gut and vaginal microbiota and endometriosis, suggesting that microbial dysbiosis could influence the pathology of endometriosis [73,74].

The variability in research outcomes likely reflects the complex nature of endometriosis as a multifactorial disease influenced by genetic predispositions, lifestyle factors, and environmental exposures.

## 5. The Genomic Landscape of Endometriosis

### 5.1. Risk Loci and Epigenetic Events Associated with Steroid Hormone Imbalance

In individuals with endometriosis, crucial physiological processes, including inflammation, immune responses, angiogenesis, and apoptosis, are dysregulated. These disruptions predominantly arise from alterations in the hormonal equilibrium between estrogen and progesterone [1]. Elevated levels of estrogen production and resistance to progesterone represent the primary hormonal changes observed in patients with endometriosis [1,52,75]. Estrogens, particularly 17β-estradiol (E2), are instrumental in supporting the survival, proliferation, and inflammatory responses of endometriotic tissues. Conversely, resistance to progesterone impedes the ability of progestins to mitigate the development and progression of endometriotic lesions [76].

In addition to the pivotal role of steroid metabolism and hormonal signaling in the pathophysiology of endometriosis, several candidate genetic loci implicated in sex steroid hormone pathways have been identified through genome-wide association (GWA) and meta-analysis studies [77,78,79]. Single-nucleotide polymorphisms (SNPs) associated with endometriosis have, for instance, been identified in or near estrogen receptor 1 (*ESR1*), also termed estrogen receptor alpha (ERα), growth regulation by estrogen in breast cancer-1 (*GREB1*), and the follicle-stimulating hormone subunit beta (*FSHB*), as well as the coiled-coil domain containing 170 (*CCDC170*) and spectrin repeat containing nuclear envelope protein 1 (*SYNE1*), which are located near *ESR1* and coexpressed with it [78].

Along with its relative, ESR2 (also known as ER beta or ERβ), ESR1, a member of the nuclear receptor family of proteins, mediates the effects of estrogen on gene expression. Specifically, ESR1 regulates genes essential for cell adhesion, proliferation, and neoangiogenesis, which are crucial for the growth of endometriotic lesions [80]. *FSHB* encodes the beta subunit of FSH, a glycoprotein hormone pivotal in ovarian folliculogenesis [81]. *GREB1*, which has been identified in previous GWASs [82,83], is a target gene of E2/ERα [84] and functions as a chromatin-bound estrogen receptor cofactor in endometriosis, promoting the growth and proliferation of estrogen-responsive cells [84,85,86,87]. CYP2C19, a member of the cytochrome p450 enzyme family, plays a significant role in drug and estrogen metabolism. Certain *CYP2C19* polymorphisms associated with increased CYP2C19 activity confer a decrease in estrogen levels, potentially reducing the risk of endometriosis and vice versa [88,89]. Moreover, risk variants in *CCDC170* and *SYNE1* may influence estrogen biosynthesis [78]. Notably, genetic variants at all of these loci are also associated with breast and ovarian cancers [90,91,92], suggesting a potential link between the mechanisms of estrogen action in endometriosis and the pathogenesis of these cancers.

Epigenetic modifications also play a crucial role in creating hormonal imbalances in endometriosis, characterized by elevated estrogen levels due to increased synthesis and reduced degradation. Genes involved in estrogen breakdown are often hypermethylated in endometriosis [93,94], leading to reduced enzyme expression and subsequently decreased estrogen degradation. Moreover, hypomethylation of the *ESR2* promoter region contributes to a significant increase in ERβ activity in endometriotic lesions [52].

Furthermore, altered histone acetylation enhances the transcription of aromatase, the enzyme crucial for estrogen synthesis [93,94]. This increase in aromatase expression in ectopic endometrial tissues significantly contributes to an increase in local estrogen production, thus promoting the growth of endometriotic lesions [93,94].

Endometriotic lesions also exhibit reduced responsiveness to progesterone, which is essential for normal endometrial turnover [93,94,95]. Progesterone resistance in these lesions is typically induced by epigenetic alterations, including aberrant DNA methylation and histone modifications. These changes lead to the downregulation of progesterone receptor (*PGR*) and progesterone-responsive gene expression [93,94].

Finally, specific microRNAs and long noncoding RNAs (lncRNAs), which typically suppress estrogen receptor expression, are downregulated in endometriosis. This downregulation results in increased estrogen receptor activity and amplified estrogenic responses in endometrial cells [93,94,96].

### 5.2. Identification of Additional Endometriosis Risk Loci

Unbiased large-scale genetic linkage and meta-analysis studies have identified several additional genomic loci with significant associations with endometriosis and confirmed previously reported associations. Most notably, the International Endogene Consortium conducted large-scale family-based linkage studies of endometriosis in cohorts of Australian and UK families. Combined cohort studies revealed two linkage regions on chromosome 10q26 [97] and chromosome 7p13-15 [98] that may harbor rare causal variants. Subsequent studies by the same consortium reported a third region of suggestive linkage located on chromosome 20p13 [97]. Subsequent genome-wide association studies conducted in women of European ancestry led to the identification of two additional risk loci that mapped to chromosomes 7p15.2 and 1p36.12 [99] and were independently confirmed by studies in Japanese and European cohorts [82,100].

Systematic gene sequencing of these risk loci (1p36.12, 7p15.2, 10q26, and 20p13) failed to causally link single-risk gene variants to endometriosis. Only the variant associated with the linkage peak on chromosome 7p13-15 has been reported to represent a susceptibility allele with high penetrance for more severe forms of endometriosis [98]. Tapmeier et al. expanded upon their previous work on this linkage signal for endometriosis by identifying and validating variants of the neuropeptide S receptor 1 (*NPSR1*) gene located within the 7p13-15 chromosome region that are associated with endometriosis in both human patients and rhesus macaques [101]. The study showed that deleterious low-frequency coding variants in *NPSR1* are more common in patients with familial endometriosis, particularly in moderate to severe stages. NPSR1 is a G protein-coupled receptor that binds to neuropeptide S (NPS) and is involved in various physiological processes, including the regulation of anxiety, stress, sleep, and nociception (pain perception).

Although the precise mechanisms by which NPSR1 might contribute to endometriosis remain to be defined, known functions of NPSR1 that may be particularly relevant to endometriosis include the regulation of immune responses and inflammation and the modulation of pain perception. *NPSR1* expression in macrophages and T lymphocytes is elevated in several inflammatory diseases, such as inflammatory bowel disease [102] and asthma [103,104]. Variants in the *NPSR1* gene could thus potentially influence inflammation and the perception of pain symptoms in endometriosis patients. Consistent with this notion, Tapmeier et al. showed that the administration of the NPSR antagonist SHA 68R significantly relieved inflammation and pain in mouse models of endometriosis [101]. Overall, these studies suggest that NPSR1 is a nonhormonal target in endometriosis.

GWASs typically require large sample sizes to detect associations with enough statistical power, especially for variants with small effect sizes. This can represent a significant limitation when identifying causal variants in polygenic diseases such as endometriosis. A recent meta-analysis of 24 genome-wide association studies, the largest to date, investigated 60,674 cases and 701,926 controls of European and East Asian descent. This analysis identified 42 genome-wide significant loci comprising 49 distinct association signals [105]. These identified signals were associated with more advanced stages of ovarian endometriosis, and alterations in the regulated expression or methylation of genes in the endometrium and blood were identified. Notably, many of the genes causally related to endometriosis risk variants were found to be linked to pain perception/maintenance, including signal recognition particle 14 (*SRP14/BMF*), ganglioside-induced differentiation-associated protein 1 (*GDAP1*), histone lysine methyltransferase DOT1L cofactor (*MLLT10*), bassoon presynaptic cytomatrix protein (*BSN*), and nerve growth factor *(NGF*). Overall, significant genetic correlations were detected between endometriosis and eleven pain-related conditions, including migraine, back pain, multisite chronic pain, and inflammatory conditions such as asthma and osteoarthritis. The identification of these genetic markers, along with *NPSR1* could, therefore, facilitate the development of novel therapeutic strategies aimed at mitigating the pain and inflammation associated with this disease.

GWAS and meta-analysis studies further identified loci associated with increased endometriosis risk, including SNPs in and around interleukin 1 alpha *(IL1A*) [106] and WNT family member 4 (*WNT4)* [99,107], encoding a protein involved in the WNT signaling pathway critical for the development of the female genital tract [82,108]. Other genes regulate cell proliferation and differentiation, cell survival and apoptosis [77,100,109], cell adhesion, migration, and invasion (such as fibronectin 1 (*FN1*) and vezatin (*VEZT*)) [82], as well as detoxification, and inflammation [77,100]. Nevertheless, many of the specific variants responsible for the observed association signals and their functional roles in facilitating the underlying disease-causing mechanisms remain to be identified.

## 6. Endometriosis as a Neoplastic Disease

### 6.1. Somatic Mutations in Cancer Driver Genes

Although considered a benign disease, endometriosis shares striking similarities with cancer. The ectopic endometrial-like epithelium and stroma [1,14] exhibit neoplastic characteristics, including resistance to apoptosis, stimulation of angiogenesis, invasion, inflammation, and spread, which are typically observed in cancer [110]. These neoplastic characteristics are mirrored by the pathways and gene networks identified in genetic linkage studies. Even more significantly, recent studies suggest that these characteristics, similar to the hallmarks of cancer, may be conferred by somatic cancer-associated mutations in endometriotic lesions.

Candidate gene mapping and linkage studies were the first to implicate tumor suppressor genes in endometriosis: loss of heterozygosity (LOH) was detected in genes encoding phosphatase and tensin homolog deleted on chromosome 10 (*PTEN*) [111], tumor protein p53 (*TP53*), and AT-rich interactive domain-containing protein 1 (*ARID1)* [112]. These findings provide a potential link between endometriosis development and malignant transformation. This association has been reinforced by next-generation sequencing studies on laser microdissected endometriotic tissues, which revealed recurrent somatic mutations in cancer driver genes [113,114,115]. The genes most commonly mutated in endometriosis patients are the genes encoding ARID1A and components of the MAPK/RAS and PI3K-Akt-mTOR signaling pathways [113,114,115].

Accordingly, Anglesio et al. [113] reported frequent gain-of-function mutations in the *PIK3CA*, *KRAS,* and *PPP2R1A* genes and loss-of-function alterations in *ARID1A*, together affecting approximately one-quarter of DIE patients subjected to comprehensive genomic analysis [113]. Other recurrently mutated genes identified in endometriosis include *TP53*, *FBXW7*, and *CTNNB1.* Although these initial results are limited by their relatively small cohort size, the findings were replicated in subsequent next-generation sequencing studies. Notably, these studies have also shown that cancer driver mutations are restricted to epithelial glands but are not detected in stromal cells of the same tissue [114,115].

### 6.2. The Role of Cancer Driver Mutations in Endometriosis Development

Although the presence of cancer driver mutations in benign endometriotic lesions is clearly nonrandom, the role of these mutations in endometriosis is still unclear. All detected cancer-associated mutations are known to control cell proliferation and survival, angiogenesis, invasion, and DNA damage repair. However, driver gene mutations have been identified at similar frequencies in endometriomas, the form of endometriosis considered the origin of endometriosis-associated ovarian cancer, and other subtypes of endometriosis where malignant transformation is exceedingly rare. Indeed, all major anatomically described subtypes of endometriosis harbor recurrent somatic cancer driver alterations [114]. Moreover, the same mutations are also found in eutopic normal endometrial epithelium and iatrogenic endometriosis, a rare complication associated with laparoscopic supracervical hysterectomy [114,115]. Indeed, neither the occurrence of cancer driver mutations nor their prevalence is specific to endometriosis. Somatic driver-like events were discovered in more than half of the histologically normal endometrial samples analyzed and are also present in other normal human tissues [116].

Multiple studies have validated a relatively modest spectrum of shared driver alterations, including recurrent activating changes in *KRAS, PIK3CA, ARID1A,* and *CTNNB1* [113,114,117,118]. The shared mutational spectrum may be influenced by the conditions required for the expansion of the endometriosis epithelium at ectopic sites under selective pressure. Thus, cancer driver mutations in endometriosis may be necessary for the growth and survival of endometriotic tissue in ectopic regions of the body and may reflect the cell-intrinsic neoplastic characteristics required for the successful establishment of endometriotic lesions.

### 6.3. Endometriosis Development: Clonal Expansion and Heterogeneity

Endometriosis development is age dependent. Cells in endometriotic lesions with cancer driver mutations often originate during the first decades of life [115]. The mutagenic burden progressively increases with age and decreases with parity. The normal endometrial epithelium typically carries one driver mutation per gland [115]. This number increases in endometriotic lesions, where 1–2 cancer driver mutations are detected [113,114,117]. In particular, *KRAS, PIK3CA,* and *ARID1* mutations are frequently discovered in histologically benign endometriotic epithelia and are thought to occur early in endometriosis development [119,120].

In an individual patient with endometriosis, the mutational profile of the endometrium is similar, albeit not identical, to that of endometriotic lesions. This resemblance suggests that the glands are the origin of the initial mutations. Normal human endometrial glands and endometriotic glands are composed of clonal cell populations [115], which indicates that each gland may derive from a common progenitor cell. Clonal expansion of epithelial cells carrying somatic driver mutations may thus lead to subsequent colonization of the epithelial lining of the endometrium, with lesions acquiring additional mutations during their development [30]. DNA-based analyses have revealed clonality within regions sampled from the same lesion and across different lesions [114,115,121]. Nevertheless, examinations of endometrial glands have also demonstrated notable heterogeneity in mutational profiles, even among glands from the same patient [115], suggesting that different lesions may harbor distinct mutation profiles and be composed of unique cell clones. The heterogeneous nature was further demonstrated by the frequent presence of mutations in *PIK3CA* in endometriotic lesions analyzed across different patients but the lack of shared *PIK3CA* mutations across multiple lesions collected from the same individuals [114]. This latter result supports the notion that somatic mutations in *PIK3CA* and other cancer driver genes may confer a selective advantage for the survival of endometrial stem/progenitor cells and their ability to establish lesions.

While heterogeneity across lesions is commonly observed, there are notable exceptions. An investigation of multiple individual epithelial glands isolated from a single endometriotic lesion of one patient revealed similar *PIK3CA* mutations in all glands examined [114]. This uniformity in mutational profiles can be explained by recent findings indicating that multiple glands may originate from a common horizontal rhizome-like glandular structure in the basalis layer [30,31]. These results suggest that progeny from a common epithelial progenitor cell situated at the base of interconnected rhizome-like glands can populate endometrial glands in more distal regions. Subsequently, vertical gland development may lead to the acquisition of additional mutations, thereby increasing heterogeneity and potentially facilitating neoplastic transformation.

Polymorphisms in the apolipoprotein B mRNA editing enzyme catalytic subunit (*APOBEC*) may further contribute to heterogeneity by catalyzing aberrant DNA cytosine deamination, which leads to chromosomal instability. A distinctive APOBEC-related mutagenesis signature has been prominently observed in endometriotic tissues but not in the normal uterine endometrium [122]. Interestingly, APOBEC activity has been linked to tumor subclone diversification and heterogeneity in several types of cancer [123]. Additionally, APOBEC-catalyzed DNA mutagenesis can enhance tumor immunogenicity by generating a high load of neoantigens, leading to increased lymphocytic infiltration of the tissue [124]. Thus, APOBEC-mediated mutagenesis may serve as a significant source of the mutational burden that influences endometriotic gland diversification and immune responses.

## 7. Endometriosis-Associated Cancers

### Genetic Basis of Endometriosis-Associated Cancers

Although only 0.3–1.6% of endometriosis cases are estimated to progress to malignant transformation, endometriosis is considered a risk factor for ovarian cancer [14,125,126,127,128]. Indeed, endometriosis is observed in 4–29% of patients with ovarian cancer [129]. In addition to ovarian cancer, endometriosis is associated with a greater risk of ovarian and thyroid cancer and a minimally elevated risk (4%) of breast cancer [130].

Ovarian cancers constitute a heterogeneous group of cancers categorized into five main categories [131,132,133,134] based on histopathological and molecular characteristics and clinical behavior: high-grade serous ovarian carcinoma (HGSC), endometrioid carcinomas (ENOC) including seromucinous borderline tumors (SMBTs), clear cell ovarian carcinomas (CCOC), low-grade serous carcinomas (LGSOC), and mucinous ovarian carcinomas (MOC). Among these subtypes, HGSC represents the most common subtype (70%). Endometrioid ovarian carcinoma (ENOC) and clear cell ovarian carcinoma (CCOC) together account for ~25% of all invasive ovarian cancers. In comparison, low-grade serous carcinoma (LGSOC) is a less common epithelial ovarian cancer type (10%) that mostly affects younger women.

ENOC accounts for the majority (75%) of EAOC cases [135,136], except for Japan, where CCOC is more prevalent, accounting for approximately a quarter of all epithelial ovarian cancers [137,138]. ENOC and CCOC represent two types of heterogeneous and understudied cancers that exhibit few similarities to the more common high-grade serous HGSOC. Patients with both ENOC and CCOC have variable or poor responses to standard platinum-based chemotherapy. CCOC, in particular, is more likely to be platinum resistant at the early stage and resistant to second-line chemotherapy at the advanced stage, resulting in worse survival.

Established endometriosis is regarded as a precursor to ENOC and CCOC [139,140,141,142]. Therefore, these cancers are also termed endometriosis-associated ovarian cancer (EAOC). Patients affected with endometriosis have a 2–4-fold increased risk of developing EAOC, and 21–51% of patients with CCOC and 23–43% of women with EAOC are also affected by endometriosis [143,144,145]. Histotype-specific associations were supported by an observational study involving 7911 women with EOC from the Ovarian Cancer Association Consortium. This study revealed a significant association between a history of endometriosis and CCOC, with an odds ratio (OR) of 3.05, ENOC, with an OR of 2.04, and LGSOC, with an OR of 2.11 [141]. A recent study analyzing large-scale endometriosis and EAOC GWAS meta-analyses confirmed and extended these associations. This study provided clear evidence of a strong genetic correlation and causal relationship between endometriosis and ENOC, CCOC, and, to a lesser extent, HGSOC and identified shared genomic regions, genetic variants, genes, and pathways that likely contribute to the causal relationship [142]. Additionally, SMBT, initially introduced in 2014 as an independent type of ovarian tumor, is now recognized in the 2020 WHO Classification of Female Genital Tumors as a variant of endometrioid carcinomas that exhibit mucinous differentiation [134]. Although not invasive and not completely benign, SMBT is strongly associated with a history of endometriosis (30–70% of cases) and can develop from the same cell type as ovarian cancers [134].

The notion that EAOC and, specifically, the OMA subtype develop primarily from endometriomas is further supported by the analysis of the mutational profiles of resected ovarian cysts at various stages of tumor progression [135,136]. These analyses provided clear evidence for a clonal relationship between endometriosis-associated ovarian carcinomas and endometriotic lesions of this subtype [141]. Activation of the PI3K/AKT/mTOR pathway plays a crucial role in the malignant transformation of tumors, and concurrent mutations in components of this pathway are also observed in ectopic endometrium and EAOC. These mutations include loss-of-function mutations in *PTEN* and gain-of-function mutations in *PIK3CA* and *PIK3R1* [146,147,148]. In addition, *PTEN* promoter hypermethylation, which results in decreased PTEN protein levels, was detected in 18 out of 30 (60%) patients with endometriosis-associated ovarian carcinoma (EAOC) [22]. Somatic mutations are found for genes encoding mitogen-activated protein kinase (MAPK) pathway components, including *KRAS*, *PPP2R1A*, and *ERBB2*, and other cancer driver genes, such as *TP53*, *FBXW7*, and *CTNNB1*. Furthermore, endometriotic lesions and lesions in EAOC harbor loss-of-function alterations in tumor suppressor genes involved in DNA repair, either by somatic mutation or by epigenetic inactivation through promoter hypermethylation. These genes include genes encoding DNA mismatch repair proteins such as MutL protein homolog 1 (*MLH1*) [148,149,150,151,152]. Promoter hypermethylation leading to decreased protein levels has also been documented in EAOC for several other tumor suppressor genes encoding Runt-related transcription factor 3 (*RUNX3*) [18], E-cadherin (*CDH1*) [20], the Ras-association domain family of gene 2 (*RASSF2*) [21], and *CDKN2A/p16* [149,153].

Thus, mounting evidence supports a genetic and causal relationship between endometriosis and specific EAOC histotypes. Mutational analysis further suggests that the progression to malignancy of endometriotic lesions may be driven, at least in part, by the progressive accumulation of mutations due to aberrant DNA repair and dysregulated epigenetic control of gene expression. However, additional research is necessary to identify the crucial molecular features determining why specific endometriotic lesions appear to progress to malignancy. Such research will necessitate the development of improved models in order to study the impacts of particular mutations or epigenetic changes on the pathogenesis of endometriosis. Moreover, a significant unresolved question is how the microenvironment influences the growth of endometriotic lesions and their potential progression to malignancy.

## 8. Novel Insights into Disease Pathology: 3D Organoids, Single-Cell Omics, and Imaging Studies

One of the primary challenges in understanding the causes of endometriosis is the complexity of the endometrium and the extensive changes that occur in multiple cell types throughout the menstrual cycle, which are regulated by ovarian hormones. Moreover, the development of certain types of endometriotic lesions has been challenging to investigate due to the small size of diseased tissues, the heterogeneity of tissues, and the critical role of the microenvironment in disease initiation and progression. The lack of appropriate in vitro model systems that closely mimic the structure and function of the endometrium has further limited our ability to study disease pathology. The emergence of 3D organoid models that use patient cells and the advent of single-cell omics technologies is expected to revolutionize our understanding of both the normal endometrium and the drivers of endometriosis development and progression. In addition, new imaging techniques, such as tissue-clearing microscopy and 3D reconstruction, will enhance our understanding of how endometriosis affects the tissue architecture of glands.

### 8.1. Organoid Models of Endometriosis

Organotypic models have been transforming our understanding of cancer diversity and how it affects precision medicine. These studies have demonstrated the ability of organoids to maintain stable representations of the genetic, proteomic, morphological, and pharmacological features of the original tumor while also providing unprecedented control over genomic and environmental conditions. Recent progress has demonstrated the potential of applying this technology to the study of endometriosis.

Several studies have recently established long-term organoid cultures with which to examine the function of the normal endometrium and its response to hormones during the menstrual cycle [154,155,156,157]. Further studies have highlighted the ability of organoid cultures to accurately and stably reproduce the intra- and interlesion biological heterogeneity found in individual endometrial lesions both within and between patients [155] and in tumor biopsies obtained from endometriosis-associated cancer tissues. Moreover, the use of organotypic models to study patient-specific ovarian tumors has already provided a platform for testing treatment responses and screening new drugs for efficacy against different ovarian cancer subtypes [158]. As such, organoids have the potential to provide standardized platforms for dissecting disease mechanisms and developing more effective treatment strategies for patient care.

### 8.2. Single-Cell Omics Approaches

Endometriosis development is characterized by intricate interactions among the endometrial epithelium, immune cells, and stroma within the endometrial microenvironment. These complex dynamics are not amenable to analysis with traditional bulk transcriptomics approaches and are inadequately represented in current 3D organoid models. However, advances in single-cell RNA sequencing and spatial transcriptomics have enabled a more detailed examination of the cellular complexity and heterogeneity inherent to endometriosis.

Recent research has underscored the effectiveness of advanced technologies in tracking the dynamic changes in the human endometrium throughout the menstrual cycle and pregnancy [159,160,161]. These studies have revealed how variations in the epithelial and stromal composition of lesions are linked to hormonal fluctuations across different menstrual phases. Such insights significantly enhance our understanding of the mechanisms and consequences of altered hormonal responses in endometriosis, particularly those related to estrogen and progesterone action.

Moreover, several studies have systematically characterized various types of endometriotic lesions, including peritoneal and ovarian lesions, by comparing these with normal endometrial or ovarian tissue [36,162,163,164,165,166,167]. In addition, Guo et al. applied mass cytometry to analyze the composition of cells within peritoneal fluid and peripheral blood from both patients and controls [168]. This research has provided new insights into the heterogeneity of endometriosis, highlighting the progressive accumulation of germline, epigenetic, and somatic mutations associated with this disease. Moreover, these studies identified an immunotolerant peritoneal niche, delineated differences between ectopic and eutopic endometrium, and elucidated variations between lesion microenvironments. Distinctive expression signatures of endometriosis, including inflammatory responses, immune tolerance, hormonal reactions, angiogenesis, and fibrosis development, have been recognized across various subtypes and cellular subpopulations of the disease [36,162,163,164,165,166,167,168].

A major finding from these studies has been the identification of dysfunctions in pro-inflammatory pathways within the endometriotic niche. This has been demonstrated by the increased presence of both innate immune cells (macrophages and dendritic cells) and activated T cells in the endometriotic lesions and peritoneal fluid of patients and the enhanced immune trafficking and upregulation of complement proteins in endometriotic tissue [36,163,165,166,168], thus underscoring the role of the inflammatory microenvironment in the development and progression of endometriosis. Furthermore, Yan et al. have shown that epithelial cells in ectopic ovarian lesions induce the upregulation of HLA class II complex expression in macrophages, which activates CD4^+^ T cells and contributes to chronic inflammation in ovarian endometriosis [164]. In contrast, regulatory T (Treg) cells, which are essential for limiting excessive inflammation, were found to be reduced in ectopic ovarian and peritoneal lesions [164,167]. A greater proportion of CD4^+^ T cells and a lower proportion of Treg cells may, therefore, stimulate the inflammatory responses of T cells in ectopic lesions.

A second notable finding from these studies has been the demonstration of immune tolerance or immune evasion [36,163]. Specifically, several studies have demonstrated that both the number and cytotoxicity of NK cells are significantly reduced in the peritoneal fluid and menstrual effluent of endometriosis patients [162,166,168]. Similarly, Ma et al. have reported a decreased proportion of cytotoxic NK cells in ectopic lesions [167]. This reduction suggests a mechanism by which menstrual fragments may survive during retrograde menstruation and persist within the peritoneal cavity. Similarly, in endometrial carcinoma, there is a preferential enrichment of exhausted CD8^+^ T cells and macrophages in tumor tissues compared with adjacent paratumor tissues [169], indicating the development of immune evasion.

Taken together, the results of single-cell transcriptomics studies have highlighted the important role of immune dysregulation in endometriosis and confirmed earlier findings [170]. While a limitation of these studies is the small sample size employed, it can be anticipated that the compilation and integration of results into databases such as the Human Endometrial Cell Atlas (HECA), an advanced single-cell and single-nucleus reference atlas, will rapidly overcome these limitations. HECA currently encompasses metadata for approximately 630,000 cells and nuclei [171]. Such consolidated datasets will enhance our understanding of the spatiotemporal organization and functionality of endometriotic microenvironments. By integrating single-cell data with genetic data, it will be possible to dissect the contributions of gene variants to endometriosis and identify crucial cell populations and signaling networks. Finally, single-cell transcriptomics data may also serve as the basis for the development of new therapeutic strategies [172].

## 9. Crosstalk between Endometriotic Lesions and the Microenvironment

While the initial discovery of cancer driver mutations in endometriotic tissues has sparked considerable enthusiasm, subsequent research has revealed that these somatic mutations alone do not fully account for the gradual progression of endometriosis to malignancy. A substantial body of evidence now demonstrates that the development and progression of endometriosis to malignancy are influenced by a range of cell-autonomous and nonautonomous factors. These include genetic predispositions, somatic cancer driver mutations, epigenetic changes, and external factors, such as lifestyle influences and microenvironmental conditions. Specifically, advancements in single-cell RNA sequencing have redirected attention toward the intricate interactions between endometriotic cells, the stromal compartment, and immune cells within the local microenvironment, which are crucial to disease pathogenesis. Below we will summarize key hallmarks of endometriosis development and progression that incorporate these factors, including (1) genetic predisposition, (2) estrogen/progesterone hormonal imbalance, (3) acquisition of cancer driver mutations, (4) inflammation, (5) immune evasion, and (6) neoangiogenesis.

Polymorphisms or gene mutations related to estrogen metabolism, cell proliferation, cell survival, and immune function can predispose individuals to endometriosis. It is hypothesized that endometriosis primarily arises from retrograde menstruation, which transports tissue fragments into the peritoneum. These endometrial lesions are likely derived from stem/progenitor cells present within these fragments, given their high regenerative potential and ability to initiate new endometrial tissue growth in ectopic locations. These progenitor cells undergo clonal expansion, forming nascent glands and establishing endometriotic tissue within the epithelium.

These progenitor cells may already exhibit a variety of molecular abnormalities due to genetic risk factors, including the altered expression of steroid receptors and aromatase, as well as the limited presence of somatic cancer driver mutations. Such abnormalities can include loss-of-function mutations or aberrant epigenetic regulation of tumor suppressor genes, such as *ARID1A* and *PTEN* or activating mutations in the PI3K/KRAS/MAPK pathway, which often manifest early in the endometrium. These changes can enhance cell proliferation and survival, thereby contributing to the establishment and growth of lesions. Over time, endometriotic cells are likely to accumulate additional alterations, predisposing them to transformation—a process further influenced by the surrounding hormonal and inflammatory conditions.

Sex hormones, in conjunction with germline and somatic alterations, play a crucial role in the initial adhesion, growth, and angiogenesis of ectopic endometriotic tissue. The E2/ERα complex is primarily involved in mediating the effects of estrogen on cell proliferation by stimulating the transcription of genes that support cell cycle progression and inhibit apoptotic pathways, contributing to the survival and expansion of endometriotic lesions [173]. The E2/ERα complex also promotes the remodeling of the extracellular matrix, which is crucial for the invasion of endometrial cells into surrounding tissues [173].

By contrast, E2, acting through ERβ, serves as a primary driver of pathological processes in endometriosis that enhance lesion survival and inflammation [93,173,174]. The E2/ERβ complex modulates the inflammatory environment by upregulating the production of inflammatory cytokines and other mediators that exacerbate pain and inflammation in affected areas. This includes the induction of cyclooxygenase-2 (*COX-2*), encoding an enzyme that further increases prostaglandin E2 (PGE2) levels, intensifying inflammation and pain sensitivity [173,174]. Elevated levels of PGE2 also stimulate the overexpression of aromatase, leading to increased conversion of androgens to estrogens, thus elevating E2 levels and enhancing signaling through ERβ [173,174]. Furthermore, PGE2 promotes angiogenesis by affecting immune and endothelial cells and regulating the production of proangiogenic growth factors such as vascular endothelial growth factor (VEGF) [173,174]. Ultimately, estrogens promote cellular survival and sustain the inflammatory response primarily through ERβ, thereby perpetuating a vicious cycle resulting in chronic inflammation that drives endometriosis development [96].

Endometriotic lesions typically also exhibit diminished responsiveness to progesterone, which normally counteracts estrogen’s action [175]. Consequently, the imbalance between estrogen and progesterone facilitates the adhesion, growth, and survival of endometriotic founder tissue.

As evidenced by numerous studies, inflammation plays a crucial role in endometriosis. Inflammatory cytokines and growth factors not only promote survival and proliferation but also assist in creating an environment that is similar to that observed during cancer development. Inflammation in endometriosis is sustained by the recruitment of macrophages and other activated leukocytes from the bone marrow to both the developing endometriotic lesions and the eutopic endometrium by locally secreted chemokines [175]. Within endometriotic lesions, recruited immune cells release high levels of proinflammatory cytokines, further enhancing the local inflammatory response and leading to progressive deregulation of sex hormone signaling. Inflammation and hypoxia also play important roles in regulating the steroidogenic pathway. Ultimately, these interactions result in the establishment of a positive, aberrantly regulated, inflammatory–hormonal feedback loop that drives ectopic endometrial growth and disease progression.

While inflammation is essential for disease development and progression, impaired immune surveillance is also crucial, allowing atypical or precancerous cells in endometriotic lesions to persist and evolve. Under normal conditions, the immune system recognizes and eliminates these cells. However, this mechanism appears to be compromised in endometriosis, as evidenced by various studies, including single-cell RNA sequencing studies, that have revealed decreased NK function, low Treg numbers, and impaired phagocytic activity by macrophages in the endometriotic niche as discussed in the previous section.

Iron-induced oxidative stress is a significant factor in the development and progression of endometriosis, leading to the generation of reactive oxygen species (ROS) [176,177]. Excess ROS production is believed to be triggered in response to erythrocytes, apoptotic endometrial tissue, and cell debris entering the peritoneal cavity during retrograde menstruation. This increase in ROS primarily arises from the activation of macrophages that degrade senescent erythrocytes, releasing prooxidant and proinflammatory factors, such as hemoglobin and its highly toxic byproducts, heme and iron, into the peritoneal environment [176,177]. Additionally, iron released during hemolysis, compounded by a defective or overloaded peritoneal iron disposal system, results in iron overload within the peritoneal environment [176,177], acting as a catalyst in the Fenton reaction and further amplifying ROS production.

ROS can induce oxidative DNA damage that causes genetic alterations [176,177] in endometriotic cells because they are directly exposed to these derivatives. In addition to causing direct oxidative damage, ROS can activate nuclear factor-kappa B (NF-κB) and stimulate the production of VEGF [176,177]. Elevated levels of NF-κB and VEGF consequently increase the production of cytokines, chemokines, adhesion molecules, and factors that promote lesion growth and neoangiogenesis.

Neoangiogenesis is essential for the growth of endometriotic lesions and, thus, for the progression of endometriosis. This process is triggered by hypoxic conditions and inflammatory signals within proliferating ectopic lesions, forming new blood vessels that provide essential nutrients and oxygen to the lesions [178]. In addition to VEGF, inflammatory cytokines, such as IL-1β, IL-6, and PGE2, play significant roles in regulating the formation of new vessels [178]. Furthermore, somatic cancer driver mutations within endometriotic cells can independently promote neoangiogenesis by disrupting normal cellular signaling and altering gene expression.

Taken together, different cell-autonomous factors interact in complex ways with microenvironmental factors to support the establishment, growth, and persistence of endometriotic lesions, contributing to the chronic nature of the condition. Long-term endometriosis is characterized by repeated cycles of tissue injury and repair, leading to the development of a highly inflammatory tissue environment that may enhance DNA damage and epigenetic modifications within ectopic endometrial cells. Over time, endometriotic cells may accumulate additional mutations due to dysfunctional DNA repair systems or other mutagenic processes. The resulting genomic instability may then facilitate cellular transformation in combination with immune evasion mechanisms and other microenvironmental factors. This makes the microenvironment of endometriosis a key target for therapeutic intervention so as to prevent endometriosis development and progression to malignancy.

## 10. Conclusions and Future Directions

Endometriosis has long been recognized as a disease that significantly impacts women’s health and fertility; however, it remains an understudied condition with a lack of efficient or permanent treatment solutions. This has mainly been due to the lack of understanding of the underlying molecular mechanisms involved. Population studies have clearly shown that endometriosis exhibits a significant hereditary component, as it is associated with a familial predisposition. However, the polygenic and multifactorial nature of the disease has made it difficult to identify the different genetic effects.

Studies performed over the last decade have begun to elucidate the pathogenesis and pathophysiology of endometriosis. Linkage and sequencing studies have identified genes and pathways important for the development of endometriosis, highlighting potential causal links between endometriosis and endometriosis-associated cancer. Signaling pathways that are frequently deregulated in endometriosis and endometriosis-associated cancer due to mutations in cancer driver genes are potential targets for cancer therapy in EAOC. These pathways include the PI3K/AKT/mTOR and MAPK pathways and their downstream signaling pathways. Different epigenetic processes also play a role in the genesis and progression of ovarian cancer, making them attractive candidates for diagnosis and targeted treatment, particularly considering the reversibility of epigenetic changes. Furthermore, the identification of novel subpopulations of stem/progenitor cells, coupled with advancements in technologies such as tissue-clearing techniques and three-dimensional optical imaging, offers a foundation for enhancing our understanding of the roles of these cells in the initial stages of endometriosis development.

Endometriosis development and progression are driven by a complex interplay of hormonal, immunological, and genetic/epigenetic factors that foster the establishment and growth of ectopic lesions. Mounting evidence highlights the role of the microenvironment in this process. The emergence of single-cell genomics applications is expected to have a transformative impact on the study of endometriosis and its progression to malignancy. Additionally, organoid systems can reproduce many characteristics of native tissues and disease states. Further innovations in 3D models and their integration with genetic model systems that better reflect interactions with microenvironmental factors will likely enable more accurate endometrial disease modeling and lead to the identification and targeting of key molecules that drive the growth and progression of endometriosis.

Although we are still far from understanding the molecular basis of endometriosis and its progression to malignancy, the results obtained thus far may benefit clinics by providing a basis for risk stratification. The identification of women with endometriosis who are at risk of cancer development provides a basis for improved diagnosis and prognosis and is likely to aid in improved cancer surveillance of patients at risk in the not-too-distant future. This is of clear clinical significance, as the occurrence of different somatic and epigenetic events may lead to earlier and improved diagnosis and prognosis (biomarkers) and offer improved targeted treatment options.

## Data Availability

Not applicable. No new data were obtained.
